# External branch spinal nerve paralysis on keloid scar

**DOI:** 10.11604/pamj.2016.24.12.9354

**Published:** 2016-05-04

**Authors:** Samia Frioui, Faycel Khachnaoui

**Affiliations:** 1 Service de Médecine Physique et de Réadaptation Fonctionnelle, CHU Sahloul Sousse, Faculté de Médecine Ibn El Jazzar, Sousse, Tunisie

**Keywords:** Spinal nerve, paralysis, EMG, surgery

## Image in medicine

The paralysis of the external branch of spinal nerve is very rare. It manifests clinically by a weakness and abnormal morphology of the shoulder. We must think about it in front of any simple surgery of the cervical region. We report the case of a 20 year old patient, who consulted several doctors for pain and progressive weakness of the left shoulder appeared a few days after resumption of a keloid scar complicating surgical excision of a cervical lipoma operated some months earlier. Physical examination revealed strength of the left shoulder listed on 3 without articular limitation, atrophy of the trapezius muscle with ipsilateral asymmetry and fall of the left shoulder. A lesion of spinal nerve was suspected and an EMG was executed. The EMG objectified a partial lesion of the left spinal Nerve. The patient was sent in Plastic and Reconstructive surgery for nerve repair. The achievement of the external branch of spinal nerve is manifested by pain and weakness in the shoulder triggered by the anteflexion movements of the upper limb. The most usual cause is cervical lymph node biopsy. In our case, the spinal nerve lesion occurred while the resumption in keloid skin scar. This is explained by the very superficial location of the Spinal Nerve.

**Figure 1:**
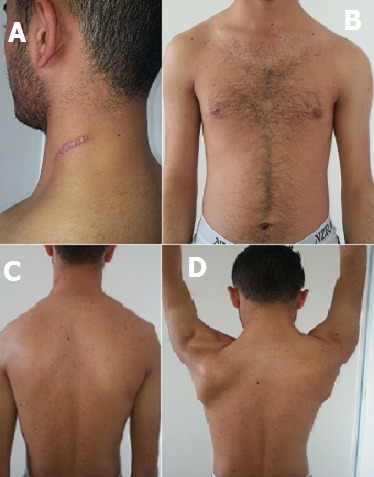
(A) scar causing paralysis of the external branch of the left spinal nerve; (B) drop left shoulder front view; (c) drop left shoulder back view; (D) peeling off of the left scapula at the shoulder antepulsion

